# Interactive voice response surveys as a method for increasing the representativeness of rural respondents in a mortality mobile phone survey: Findings from Malawi

**DOI:** 10.1111/tmi.70005

**Published:** 2025-07-10

**Authors:** Malebogo Tlhajoane, Funny Muthema, Michael Chasukwa, Kelly McCain, Shammi Luhar, Julio Romero Prieto, Jacob Saikolo, Cremildo Manhica, Sarah Walters, Boniface Dulani, Georges Reniers

**Affiliations:** ^1^ Department of Population Health Faculty of Epidemiology and Population Health, London School of Hygiene and Tropical Medicine London UK; ^2^ Department of Clinical Research Faculty of Infectious and Tropical Diseases, London School of Hygiene and Tropical Medicine London UK; ^3^ Institute of Public Opinion and Research Zomba Malawi; ^4^ Department of Politics and Government University of Malawi Zomba Malawi; ^5^ Afrocentric Governance of Public Affairs, Faculty of Humanities North‐West University Potchefstroom South Africa; ^6^ Department of Infectious Disease Epidemiology Imperial College London London UK; ^7^ Department of Survey and Health Observation National Institute of Health Maputo Mozambique

**Keywords:** interactive voice response, mobile technology, rural populations, survey sampling

## Abstract

**Objectives:**

Our study aims to (i) evaluate the yield and costs of a fully automated interactive voice response survey as a screening tool for identifying rural respondents for participation in a mortality mobile phone survey, and (ii) compare mortality survey call outcomes among interactive voice response pre‐screened and unscreened numbers.

**Methods:**

In order to identify respondents living in rural areas, a short interactive voice response survey was conducted among 24,924 unique mobile phone numbers to determine place of residence (Rural vs. Other). We calculated the proportion of rural numbers derived from the interactive voice response survey among all numbers dialled. Mobile phone numbers screened with interactive voice response were then combined with those generated via random digit dialling and used in a national mortality mobile phone survey in Malawi. Final dispositions for each mobile number dialled were compared for both groups by testing the difference in proportions.

**Results:**

Approximately half of all phone numbers dialled in the interactive voice response survey were answered, and among them, 33.9% indicated that they lived in a rural area. The cost per completed interactive voice response was US$8.75 and just under half of the numbers screened by interactive voice response later resulted in a completed mortality mobile phone survey, at a cost of US$17.4 per completed mortality survey. In comparison, less than a quarter of the numbers that were not screened through interactive voice response resulted in a completed mortality survey (45.3% vs. 22.3%, *p* <0.001). On average, 12 call attempts were required to complete a mortality survey interview in the unscreened group, compared to 6.3 call attempts among the interactive voice response pre‐screened numbers.

**Conclusions:**

Interactive voice response surveys can be used to increase the representation of rural respondents in mobile phone surveys at an acceptable cost. Modifications to the interactive voice response survey process (e.g., survey timing and number of call attempts) should be explored further to increase engagement.

## INTRODUCTION

The increased use of mobile phones in low‐ and middle‐income countries has provided new opportunities for rapid survey data collection [1, 2, 3]. In sub‐Saharan Africa alone, there were an additional 59 million mobile phone subscribers between 2018 and 2021 [4, 5], with a further projected rise of 98 million (19%) by 2025 [5]. Digital technologies are increasingly used in healthcare, either through the embedded use of mobile phones in health screening and service delivery [6, 7], or for data collection through mobile phone surveys [8, 9, 10]. Mobile phone surveys can be conducted at a relatively low cost and do not require in‐person contact, and are therefore an appealing alternative or complement to conventional household surveys (e.g., Demographic and Health Survey (DHS) and the Multiple Indicator Cluster Survey (MICS)) [11, 12, 13]. The utility of mobile phone surveys became evident during the COVID‐19 pandemic when enumerator mobility was restricted [14]. They also have applications in settings where there is political instability or in humanitarian emergencies. One example is in northern Cameroon where mobile phone surveys were used during a conflict to conduct rapid health assessments in the most afflicted areas [15].

Various methods can be used to constitute a sample for a mobile phone survey. These include: (i) sampling individuals from an existing frame such as a census or representative survey where mobile phone numbers have been collected (e.g., MICS plus [10]), (ii) obtaining phone numbers directly from telephone operators who may be able to identify numbers fitting certain criteria (i.e., by geographic location of use), and (iii) through random digit dialling [16, 17]. Where other methods are not available, random digit dialling is the default method.

While random digit dialling provides an easy way to develop a sample for a mobile phone survey, this tends to be resource‐intensive, given that enumerators will have to dial numbers that are not in operation, unregistered or belonging to respondents who are not eligible [18]. In addition, it is often necessary to set quotas for different population strata to ensure that the resulting sample is balanced in terms of key background characteristics [19]. Filling quotas for population subgroups with low mobile phone ownership can be particularly time consuming. Malawi is an interesting case study in this regard because mobile phone ownership is relatively low compared to other countries in Sub‐Saharan Africa. Data from the 2015 to 2016 Malawi DHS indicated that approximately 54% of households owned a mobile phone [20]. Within this number, important regional and gender differences were found as 86.2% of urban households reported mobile phone ownership, compared to only 47.8% of those in rural areas where approximately 84% of the population resides [20, 21, 22]. In addition, the gender divide in mobile phone ownership in Malawi is also large: 53% of men reported mobile phone ownership, compared to just 33% of women. This gender difference was even more pronounced in rural areas [20].

This study is comprised of three primary objectives: (1) to evaluate the yield (proportion of rural numbers identified) and survey completion rates of an automated interactive voice response survey (IVR) as a screening method to identify rural populations in Malawi; (2) to calculate the costs associated with pre‐screening phone numbers through the IVR, prior to conducting a national mortality mobile phone survey; and (3) to compare call attempts, call outcomes and call completion rates following a mortality mobile phone survey between phone numbers that were pre‐screened through IVR, to those that were unscreened.

## METHODS

### Study setting and survey implementation

This study was nested in the Malawi *Rapid Mortality Mobile Phone Surveys* (RaMMPS) project. RaMMPS is a multi‐country collaborative project which seeks to develop methods and tools for measuring mortality in countries where Civil Registration and Vital Statistics are incomplete [23]. The Malawi RaMMPS targeted men and women aged 18–64 years. Survey questions for the mortality survey were adapted from standard instruments routinely used for generating mortality estimates in surveys and censuses. In the case of Malawi RaMMPS, this included a series of questions on the respondent's background characteristics, followed by four modules eliciting data on household deaths, sibling and parental survival histories, and birth/pregnancy histories. Lastly, data were collected on COVID‐19 vaccinations and perceptions.

Given the differences in mobile phone ownership in Malawi, and to ensure representativeness, we imposed quotas using the 2018 national census distribution of the population by sex, age, region and urban/rural place of residence (Table S1) [21]. Quota targets for RaMMPS Malawi were set with the goal of reaching a total sample size of 20,000 participants, organised into four block periods of data collection lasting between three and five months, each with a target of 5000 completed Computer Assisted Telephone Interviews (CATIs). Quota targets were reset at the start of each data collection period.

#### Random digit dialling for sample constitution for a national mortality mobile phone surveys

The sampling frame for RaMMPS Malawi was derived through ‘screened’ random digit dialling, implemented by a third party operator (Sample Solutions) [24]. This consisted of a three step process. First, mobile phone numbers were generated by drawing a simple random sample following the local numbering structure of mobile phone providers in Malawi. Second, non‐valid numbers were removed using data from the International Telecommunication Union, and a sample was drawn proportional to the market share of the three mobile phone providers in Malawi‐ GAIN Ltd.: 4%, TMN: 42%, Zain Malawi: 54%. Finally, mobile phone numbers were verified against the Home Location Register, a database containing information regarding authorised subscribers using a global system for mobile communication core network. An estimated 80% of the numbers derived this way were thought to be operational [24]. Although use of the third party operator came at an additional cost, this reduced the calling load for RaMMPS survey enumerators who would otherwise have to dial numbers that are not in operation.

The set of numbers provided by the third party operator was not disaggregated by any of the attributes of the subscribers, hence the need to set quotas for pre‐defined strata. To that end, the CATI interview started with a number of eligibility screening questions (e.g., age, gender, place of residence). Whenever a quota in a fieldwork block was filled, it was locked, and any mobile numbers for eligible participants were banked and recalled at a later stage for the mortality mobile phone survey. This approach has been described elsewhere as random digit dialling with active strata monitoring [19]. Whenever a quota was open, the CATI interview proceeded with the consent statement.

### Mortality mobile phone survey implementation

RaMMPS CATI interviews were undertaken using the SurveyCTO platform for conducting telephone interviews. The survey employed 15 enumerators who had a minimum diploma qualification. Enumerators worked from home and calls were placed daily, including on weekends if this was convenient to the participant. In order to ensure confidentiality and maintain privacy, calls were made from a private area in the home using headsets. If a respondent requested a callback during the introductory phase of the call, the call outcome was recorded as deferred, and a subsequent attempt was made at a time convenient to the respondent. In some instances, where a household member was recommended for participation in the mortality survey by another household member, the initial call attempt was recorded as a referral and the interview proceeded for the referred individual. Interviews were monitored by the data manager and project manager daily. This included re‐calling a random subset of the respondents, to confirm that the interview had been conducted and verify some of the information collected. A random selection of the interviews (~3%) were also recorded and recordings monitored for data verification purposes. The final disposition (case outcome) of the CATI for each mobile phone number was recorded after five attempts. A detailed description of call outcomes and their definitions is provided in Table S2.

### IVR screening survey

In addition to the screened RDD, we developed and fielded an automated IVR survey to identify and augment the representation of rural respondents in the mortality mobile phone survey. A script was created for the IVR survey with two questions to determine the respondents' preferred language of communication and their place of residence, that is, urban/rural (Table S3). As outlined in the Malawi 2018 national census, urban areas within the country refer to the four major cities—Blantyre, Lilongwe, Mzuzu and Zomba, as well as Boma's (district headquarters or other urban area) and gazetted town planning areas [21]. As a result, rural residence for the IVR was determined as all those who did not reside in a city, Boma or town planning areas.

Audio recordings of the IVR script were created in each of the four main languages spoken in Malawi—Chichewa, Chisena, Chiyao, Chitumbuka. The introductory phrase for the IVR was delivered in Chichewa; the language spoken by the majority of the population. This was followed by a question which was also delivered in Chichewa to determine the language preference for the remainder of the IVR survey. Once the respondent had selected their preferred language from the four options, the remainder of the survey was conducted in the selected language. IVR calls cost US$0.59 per minute, however no charges were incurred by the participant. The survey was administered through the engage SPARK web‐based platform [25]. IVR calls were made during weekdays and on Saturdays between the hours of 09:00 and 18:00 (local time). Where a call was unanswered, repeated attempts were made 15 min, 1 h, and 24 h after the last call attempt. If all calls to a specific mobile phone were still unanswered after the first four call attempts, then the phone number was called again at a later stage for an additional round of four call attempts, following the same calling schedule. Calls were placed using a local number in order to increase engagement rates.

### Data management and analyses

Data from all call attempts of the IVR survey were combined and assessed for each unique mobile phone number that was called during the IVR. An automated variable was created by the IVR system to indicate whether a call attempt had been answered. In instances where this information was missing, calls were assumed to have been ‘answered’ if the call duration was greater than 0 s. The final outcome for each number during the IVR are reported as (i) the proportion of unique numbers that had been answered, (ii) the proportion of IVRs that were completed, and (iii) were rural respondents (total yield), among all the numbers dialled. We then report the total duration and cost for the IVR survey per number, and the estimated costs per identified rural respondent. The latter is done by combining the costs incurred for all IVR calls to a specific mobile phone number to generate a total cost of the IVR for each number.

IVR data were merged with data from the RaMMPS CATI interviews using mobile phone number as the identifying variable. Any phone number that had been pre‐screened and identified as belonging to a rural respondent during the IVR process but was not later included in the CATI interviews was excluded from further analyses. Specific methods for this merging process are described in greater detail in the Supplementary Materials S4.

Call attempts and CATI outcome status were compared for respondents with mobile phone numbers pre‐screened using the IVR method, and those that were unscreened. Hypothesis testing for the difference in proportions in call outcomes was achieved using the z‐test in Stata 18 for each call outcome independently, with two‐tailed *p*‐values reported.

### Ethics statement

The study was granted ethical approval by the Malawi National Health Sciences Research Committee (NHSRC Ref: 22/05/2918) and London School of Hygiene and Tropical Medicine Ethics Research Committee (Ref: 26393).

## RESULTS

### IVR survey completion

**FIGURE 1:**
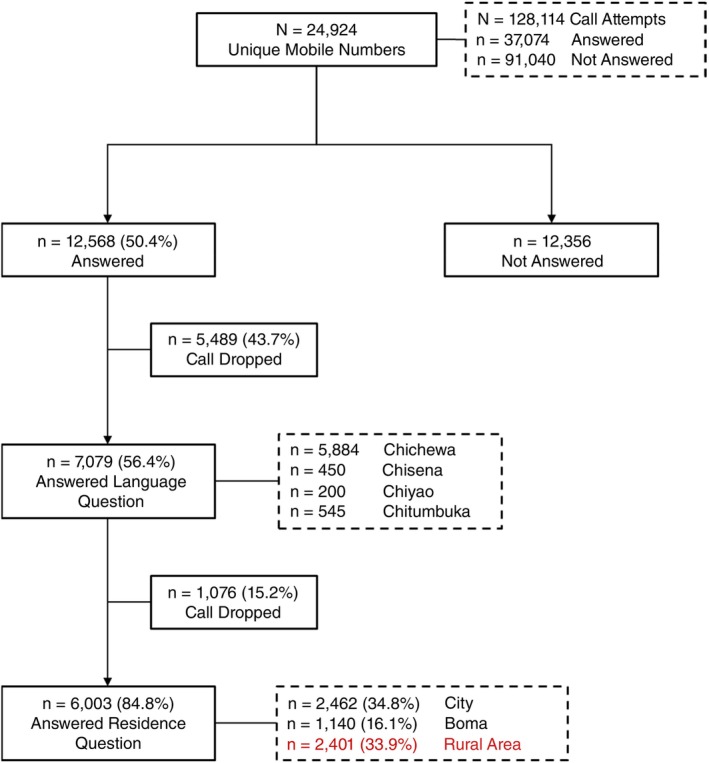
Yield of IVR survey seeking to identify rural respondents for a mortality mobile phone survey in Malawi.

**FIGURE 2:**
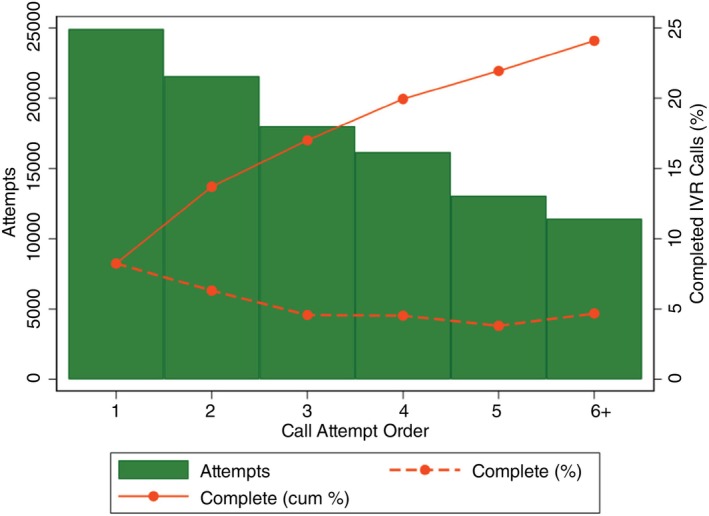
Interactive voice response survey completion among mobile phone numbers in Malawi.

IVR surveys were conducted from the 7 October 2022 to the 05 July 2023. In total 24,924 mobile phone numbers were included in the IVR survey. Overall, 12,568 (50.4%) of phone numbers were answered after all IVR call attempts (Figure 1). Of the respondents who answered the call during the IVR survey, 7079 (56.4%) went on to answer the question indicating preferred language of correspondence. This was taken as evidence of engagement with the survey. Among them, the vast majority spoke Chichewa (83.1%). The proportion of respondents who completed the IVR survey among all mobile phone numbers dialled—that is, those who answered the question on their place of residence—was 24.1%. The first two call attempts yielded the highest completion rates (8.2% and 6.3%, respectively). From the 3rd call attempt onwards, the completion rates were around 5% (Figure 2).

Overall, among the respondents who answered the question on place of residence, just over 33.9% were found to live in a rural area compared to 84% as reported in the 2018 national census [21].

### RaMMPS CATI outcomes

**FIGURE 3:**
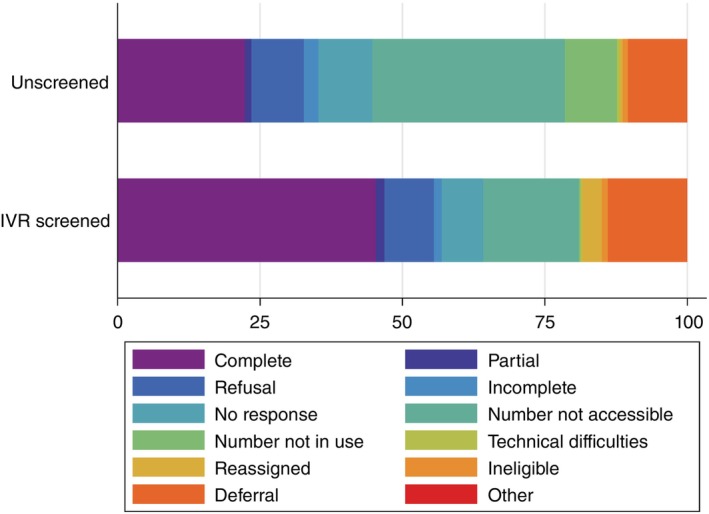
Final dispositions (final CATI call outcomes) for mortality mobile‐phone survey among unscreened and IVR pre‐screened mobile phone numbers in Malawi.

RaMMPS Malawi survey interviews began on the 24 January 2022 through 28 June 2023. Figure 3 compares the final disposition or CATI case outcomes for IVR screened and unscreened numbers. Just under half of the IVR‐screened numbers resulted in a ‘completed’ interview, compared to 22.3% of unscreened numbers (Figure 3, Table S5) (*p* <0.001). Conversely a larger proportion of the unscreened numbers were not accessible or not in use at the time of the CATI interview, compared to those that had been pre‐screened through IVR (Table S5).

**FIGURE 4:**
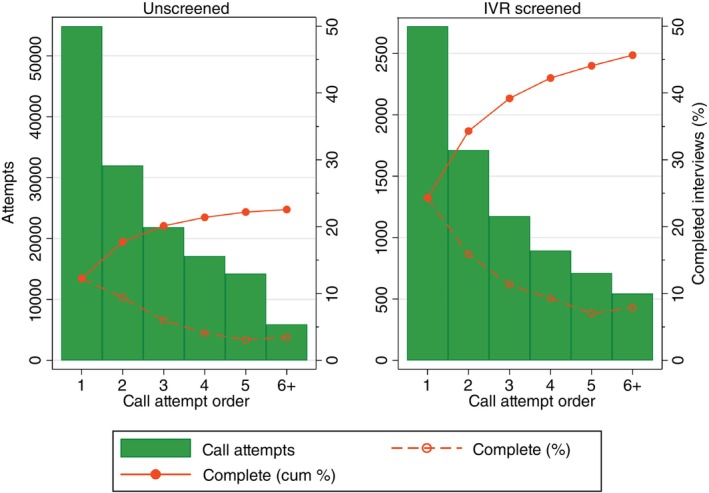
Call attempts and CATI completions for IVR screened and unscreened mobile phone numbers, by call attempt order (Malawi RaMMPS).

In total, 1209 mobile phone survey interviews were completed using numbers derived from the IVR survey. Figure 4 compares the proportion of completed CATIs in the two groups, by call attempt order. In both instances we found that most CATI completions occurred during the first two call attempts and declined with call attempt order. CATI completion was consistently higher for IVR screened numbers and remained at or above 10% even for higher order call attempts (e.g., over 2 call attempts). In contrast, the CATI completion rates had already dropped below 5% at the 4th call attempt for the unscreened mobile phone numbers, which suggests that higher order call attempts are not cost‐effective for numbers that were not screened.

Table 1 provides the (i) IVR screening costs and (ii) the costs of the IVR‐screening, per completed CATI interview. The total cost for the IVR survey was US$21,007.18, with a total mean duration of 46.0 s per number dialled. The total cost of the IVR per rural respondent identified was US$8.75, and US$17.4 per completed CATI (Table 1).

**TABLE 1:** Yield and costs of IVR survey seeking to identify rural respondents for a mobile phone survey in Malawi.

	Targeting rural residents
Unique mobile numbers	24,924
Respondents living in a rural area	2401
Completed CATI	1209 (45.0%)
Mean duration per phone number (s)	46.0
Total IVR cost (US$)	21,007.18
Cost per rural number identified through IVR (US$)	8.75
Cost per completed CATI (US$)	17.4

In Table 2, we compare estimates of the number of call attempts per completed CATI interview among mobile phone numbers screened through the IVR process to estimates of those that were unscreened. A total of 153,862 CATI call attempts were made in the RaMMPS mortality mobile phone survey, 95% of them among unscreened numbers. The 153,862 attempts resulted in a total of 13,449 completed mortality surveys – 52.8% of whom were male respondents, 47.2% female. In total 11.4 call attempts were made per completed CATI interview. This was far lower among IVR screened numbers, compared to unscreened numbers (6.3 vs. 11.9).

**TABLE 2:** CATI call attempts, completed interviews and calls per completed interview, by mobile phone number screening.

Phone number screening	Calls attempts	Completed CATI interviews	Call attempts per completed CATI
Unscreened	146,237	12,240	11.9
IVR screened	7625	1209	6.31
Total	153,862	13,449	11.4

## DISCUSSION

The growing number of mobile phone subscriptions across sub‐Saharan Africa provides an emerging opportunity for using mobile phone surveys for collecting demographic and health data [5, 26]. As mobile phone ownership across the continent continues to grow, several considerations will determine how best to harness the increased opportunity of mobile phone surveys. Constituting representative samples is an important consideration in all surveys but can be particularly challenging for phone surveys in settings where mobile phone ownership is low and possibly correlated with some of the outcomes of interest. In the Malawi RaMMPS, we experienced difficulties recruiting rural respondents (especially women living in rural areas) and piloted an automated IVR survey to aide in filling sampling quotas for these respondents and reduce the burden on enumerators.

The proportion of respondents who completed the IVR survey among all mobile numbers dialled was 24.1%. Encouragingly, this was found to be higher, or comparable to, estimates reported from other studies in Southern Africa. In one example from Tanzania, researchers reported a response rate of 0.8% (fully or partially completed interviews) in an IVR survey that sought to estimate the national prevalence of tobacco use in the country, with numbers derived through random digit dialling [27]. Higher IVR response rates have been reported in other studies across Sub‐Saharan Africa however, including in Ghana (29% fully or partially completed interviews as a proportion of all numbers dialled) [16], and Uganda (>40% across all study arms) [28]. Overall, findings from the region suggest that IVR technologies are a useful and acceptable technology that can be used to reach a large number of people, at a reasonable cost [29, 30, 31]. In our study, the cost of the IVR was 0.59c per second (mean call duration of 46 s) and just under US$18 per completed CATI. While this may be an additional cost alongside those accrued from the mortality mobile phone survey interviews, the IVR proved to be useful in reducing the number of call attempts needed to complete a CATI interview (6.3 vs. 11.9), thus reducing the time that enumerators spent on the phone, screening participants. We found that mobile phone numbers that had been pre‐screened through IVR were more likely to be functional and resulted in a significantly higher proportion of completed CATIs, compared to numbers that had not be pre‐screened.

Although mobile phone coverage has been increasing in many low and middle‐income countries, ownership remains low among women in particular [32, 33]. Disparities in mobile phone ownership between men and women were highly evident within our study population, as we found that approximately a quarter of IVR respondents were women (Figure S6). This finding is compatible with results reported in other settings on the gender gap in mobile phone ownership [34, 35, 36], with one study further reporting differences in ownership across different ages, declining from age 40 upwards [36]. This gender divide poses a particular issue in studies where there is a need to target or ensure representation of women in mobile phone surveys.

Our study provides robust scientific evidence of the potential use of IVRs as a pre‐screening method for reaching groups that are generally under‐represented in mobile phone surveys, and in instances where mobile phone ownership is unevenly distributed. It is worth noting a number of limitations, however. In this instance, IVRs did not address the issue of sample selectivity by other characteristics, such as wealth and other household economic variables. As a result, additional statistical methods such as weighting were needed when analysing data from the mortality mobile phone survey to alleviate the potential for selection bias which has been documented previously in mobile phone surveys [37]. Secondly, the initial greeting message from the IVR audio recordings was delivered in Chichewa. Although this language is widely spoken in Malawi (>80% of the population), there are some respondents who may not have understood the IVR greeting message and may not have been able to engage with the survey. Research by Labrique et al. further suggested that different introductory messages, by gender and valence (informational vs. motivational messages) could affect survey participation rates for an IVR [28]. In this instance, although the introductory message was recorded by a female, we did not investigate the effect of different strategies in delivering the introductory message on our participation rates. IVR surveys were conducted in a series of 8 rounds, each with a varying number of mobile phone numbers dialled. Including an equal number within the groups might have allowed us to better compare the yield. We also varied the time between completion of the IVR call attempts and inclusion of the mobile phone numbers into the SurveyCTO system for CATIs. This meant that some respondents would have received a call from a CATI enumerator a few days after completing the IVR, while others would have had to wait over a week. Additional analyses would be useful to better understand whether there is a correlation between the time elapsed since IVR completion, invitation to participate in the CATI interview, and CATI response rates.

Our findings suggest that IVR surveys offer a viable strategy for identifying hard‐to‐reach populations in mobile phone surveys where the sampling is conducted via random digit dialling. In countries like Malawi, where mobile phone ownership is so heavily skewed towards males and people living in urban areas, the IVR approach could be used in tandem with intra‐household referrals whenever the person answering the phone is not eligible him or herself [38]. Previous studies on the use of these strategies for improving sample representativeness have reported that referrals, rather than direct dialling were useful in reaching more vulnerable groups, such as younger women, those living in areas with low connectivity, and within poorer households [39]. The synergistic effect of these strategies could be useful in the context of Malawi where the fulfilment of target quota for women was low across all enumeration areas.

## CONCLUSION

Findings from this analysis suggest that an IVR approach may be a viable strategy to identify rural and hard‐to‐reach respondents in mobile phone surveys that draw their sample through random digit dialling. This is particularly significant, given the increasing use of mobile phone surveys. Although additional resources are needed to run an IVR survey, these are offset by a reduction in the time required by CATI enumerators as evidenced by (i) the significantly higher proportion of IVR pre‐screened numbers resulting in a completed CATI interview, and (ii) the reduced number of calls per completed CATI interview among the IVR pre‐screened group compared to unscreened numbers.

## FUNDING INFORMATION

This study was made possible with financial support from the Bill and Melinda Gates Foundation (INV‐023211). The funder had no role in study design, data collection, data analysis, data interpretation, or writing of the report.

## CONFLICT OF INTEREST STATEMENT

The authors declare no conflicting interests.

## CONSENT

All participants provided oral consent for the survey, including consent for storing anonymized data in a public repository and consent to audio record the interview.

## Supporting information


**Data S1:** Supporting Information

## Data Availability

Malawi RaMMPS data can be requested from the DataFirst Repository [40], or through email to the corresponding author. Code to reproduce the results is available from the GitHub repository https://github.com/rammps-lshtm.
