# Supplementary material for: Interactive voice response surveys as a method for increasing the representativeness of rural respondents in a mortality mobile phone survey: Findings from Malawi
Source: Trop Med Int Health. 2025 Jul 10;30(9):937–45. doi: 10.1111/tmi.70005 (PMC12401643; doi:10.1111/tmi.70005)

**Supplementary Materials**

**Table S1:** Target quota set for the Malawi Rapid Mortality Mobile Phone Surveys project, disaggregated by age, gender and place of residence.

|  | **Male** | | **Female** | | **Total** |
| --- | --- | --- | --- | --- | --- |
| Age Group | Urban | Rural | Urban | Rural |  |
| 18 – 49* | 1,020 (5.1%) | 4,972 (24.9%) | 2,040 (10.2%) | 9,944 (49.7%) | 17,976 (89.9%) |
| 50 – 64 | 168 (0.8%) | 844 (4.2%) | 168 (0.8%) | 844 (4.2%) | 2,024 (10.1%) |
| Total | 1,188 (5.9%) | 5,816 (29.1%) | 2,208 (11.0%) | 10,788 (53.9%) | 20,000 (100%) |

* Women of reproductive age (18-49 years) were oversampled 1:2 to ensure sufficient power for estimating under-five mortality.

**Table S2:** RaMMPS mortality mobile phone survey final disposition definitions after five call attempts over a period of five days.

| **Outcome Category** | | **Description** |
| --- | --- | --- |
|  | Completed interview | Fully completed mortality survey interview |
|  | Partial | Partially completed mortality survey interview, no call back required |
|  | Refusal | Respondent refused to participate |
|  | Incomplete | Incomplete mortality survey interview after 5 call attempts. To be called back |
|  | No response | Busy tone |
|  |  | No answer |
|  |  | Respondent not available |
|  |  | Answered but not by mobile phone owner |
|  | Number not accessible | Number not accessible at the time of call attempt |
|  | Number not in use | Number not in use |
|  | Technical difficulties | Respondent incomprehensible |
|  | Reassigned | Case reassigned or to another enumerator (i.e. due to language or gender preference of respondent) |
|  |  | Referral |
|  | Ineligible | Already interviewed |
|  |  | Ineligible for participation |
|  | Deferral | Quota full at the time of call attempt |
|  |  | Deferred for participation at another time (e.g. following trimester) |
|  | Other | Any other call outcome |

**Table S3:** Interactive Voice Response Survey Script

| Question Number | **Category** | **Voice Prompt** | **Response Categories** |
| --- | --- | --- | --- |
| -- | Greeting Message | Hello, we are calling you from the Institute of Public Opinion and Research and are conducting a short research project on the impact of COVID-19 in Malawi. We would first like to ask in which language we can best communicate. | |
| Q1 | Language filter | Please have a look at the numbers on your telephone, and press …. If you wish to proceed in …… | 1 = Chichewa  2 = Chisena  3 = Chiyao  4 = Chitumbuka |
| -- | Project Information | For this research project, we are looking for people living in rural areas to participate. Your number has been chosen by chance. | |
| Q2 | Area of Residence | Would you say the place you usually live is a city, a boma or elsewhere? Press 1 for city, press 2 for boma, press 3 for elsewhere. | 1 = City  2 = Boma  3 = Elsewhere (Rural) |
| -- | Goodbye Message | “Thank you for your time. A member of our team will contact you at a later stage to participate in a 20-minute survey on the impact of COVID-19.” | |
| -- | Error Message | “That wasn’t a valid reply, please try again” | |
| -- | Ineligible Message | “Thank you very much for your time. Unfortunately, you don’t fit the criteria for our survey at this time. Thank you for your participation. Goodbye” | |

**Supplement S4: RaMMPS Mortality mobile phone survey data cleaning and management**

Raw data from the RaMMPS Malawi mortality survey interviews was downloaded from the SurveyCTO platform and evaluated for duplicate reports by (i) ‘caseid’, (ii) phone number and (iii) interview start time. The ‘caseid’ variable was created by the data collection team to uniquely identify individuals contacted to participate in the mortality mobile phone survey. In some instances however, multiple individuals shared the same ‘caseid’ due to a referral within the household where the initial interview was not completed. In other instances, a specific ‘caseid’ was linked to multiple phone numbers if an individual asked to be contacted at a later date on a different number. Therefore, in order to uniquely identify individuals within the mortality mobile phone survey data, a new ‘caseid’ variable was created by making two modifications. Firstly, in instances where the ‘caseid’ variable included multiple people within a household, a subscript was included to indicate this inclusion of a second individual to the same ‘caseid’. Secondly, where the same individual had multiple phone numbers on file and where at least one of these phone numbers had gone through IVR screening, they were included in the IVR pre-screened group.

Given that in some instances, household members may have been referred for participation in the mortality mobile phone survey, and shared a ‘caseid’ with the primary respondent, we sought to evaluate any instances where certain demographic characteristics changed. This was done to allow for us to subscript the ‘caseid’ variable to differentiate respondents in the household. We began by sorting the data by ‘caseid’ and the interview start time. We then flagged any observations within a given ‘caseid’ where either the respondents gender, age or phone number changed. This was limited only to non-missing values of each of the three variables. The table below illustrates the number of unique cases where at least one of these variables changed .

|  | Variable of interest | Number of cases during which a change occurred | Proportion of cases |
| --- | --- | --- | --- |
| 1. | Gender | 324 | 0.6 |
| 2. | Age | 1,273 | 2.3 |
| 3. | Phone Number | 1,556 | 2.8 |
| At least one of the three | | 2,544 | 4.5 |

Subscripts to the ‘caseid’ variable were created by adding an ‘_n’ to the end of the existing ‘caseid’ variable. Changes were made under the following conditions:

1. **Change in recorded gender:** In all instances where the gender of the respondent changed, a subscript was created to uniquely identify the two respondents. The subscripted ‘caseid’ variable was extended to all observations after the recorded change. In addition, the call outcome for the call outcome immediately before the recorded change in gender was marked as a referral, with the exception of instances where it had been recorded as (i) a completed interview, (ii) already interviewed, (iii) quota full (available/defer), or (iv) quota full (ineligible) (table S2).
2. **Change in recorded age:** Where the age of the respondent changed by a given ‘caseid’, we calculated the difference in reported ages in years and flagged all instances where the difference was greater than 5 years.
   1. Where the difference in age was more than 5 years, a subscript was added to the ‘caseid’ variable and the call outcome immediately before the recorded change was marked as a referral following the same exclusions as described above.
   2. Where the difference in age was less than 5 years, no amendments were made.
3. **Change in recorded phone number:** Where the phone number changed for a given ‘caseid’ and no changes in gender or age were recorded, the call outcome for the observation immediately before the recorded change in phone number was recorded as a deferral, following the same exclusions as described above (i.e. except where it had been recorded as (i) a completed interview, (ii) already interviewed, (iii) quota full (available/defer), or (iv) quota full (ineligible) (table S2).

**Table S5:** Mortality mobile phone survey final dispositions for unscreened and IVR screened individuals, reporting a p-value for the difference in proportions.

| **Mortality survey call Outcome** | | **IVR Screened** | **Unscreened** | **p-value** |
| --- | --- | --- | --- | --- |
|  | Complete | 1,209 (45.0%) | 12,240 (22.3%) | <0.001 |
|  | Partial | 41 (1.5%) | 643 (1.2%) | 0.12 |
|  | Refusal | 233 (8.7%) | 5,053 (9.2%) | 0.28 |
|  | Incomplete | 36 (1.4%) | 1,397 (2.6%) | <0.001 |
|  | No Response | 195 (7.3%) | 5,209 (9.5%) | <0.001 |
|  | Number not accessible | 447 (16.8%) | 18,535 (33.8%) | <0.001 |
|  | Number not in use | 7 (0.3%) | 5,031 (9.2%) | <0.001 |
|  | Technical difficulties | 9 (0.3%) | 271 (0.5%) | 0.23 |
|  | Reassigned | 91 (3.4%) | 255 (0.5%) | <0.001 |
|  | Ineligible | 29 (1.1%) | 527 (1.0%) | 0.59 |
|  | Deferral | 371 (13.9%) | 5,693 (10.4%) | <0.001 |
|  | Other | 1 (0.0%) | 15 (0.0%) | 0.77 |

**Figure S6: Results from IVR survey targeting rural female participants, fielded to 600 unique mobile phone numbers**


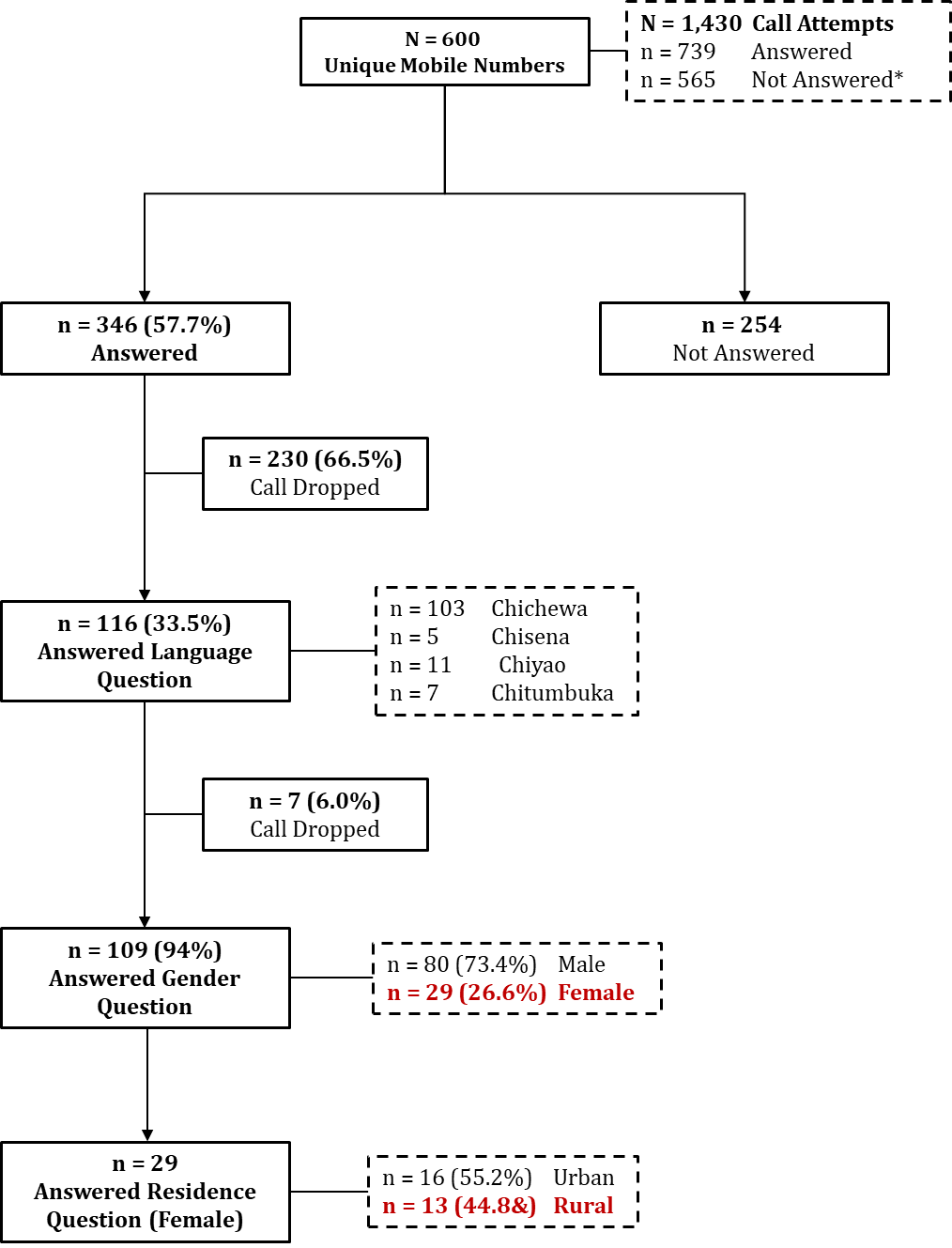

Supplement: Supplementary file 1 — Data S1: Supporting Information [file TMI-30-937-s001.docx]
